# Non-Structural Proteins of Arthropod-Borne Bunyaviruses: Roles and Functions

**DOI:** 10.3390/v5102447

**Published:** 2013-10-04

**Authors:** Saleh Eifan, Esther Schnettler, Isabelle Dietrich, Alain Kohl, Anne-Lie Blomström

**Affiliations:** 1 Department of Botany and Microbiology, King Saud University, P.O. Box 2455, Riyadh 11451, Saudi Arabia; E-Mail: seifan@ksu.edu.sa (S.E.); 2 MRC-University of Glasgow Centre for Virus Research, 8 Church Street, Glasgow G11 5JR, Scotland, UK; E-Mails: esther.schnettler@glasgow.ac.uk (E.S.); isabelle.dietrich@glasgow.ac.uk (I.D.); alain.kohl@glasgow.ac.uk (A.K.); 3 Department of Biomedical Sciences and Veterinary Public Health, Swedish University of Agricultural Sciences, P.O. Box 7028, Uppsala SE-750 07, Sweden

**Keywords:** arbovirus, bunyavirus, non-structural protein, host response, virus replication

## Abstract

Viruses within the *Bunyaviridae* family are tri-segmented, negative-stranded RNA viruses. The family includes several emerging and re-emerging viruses of humans, animals and plants, such as Rift Valley fever virus, Crimean-Congo hemorrhagic fever virus, La Crosse virus, Schmallenberg virus and tomato spotted wilt virus. Many bunyaviruses are arthropod-borne, so-called arboviruses. Depending on the genus, bunyaviruses encode, in addition to the RNA-dependent RNA polymerase and the different structural proteins, one or several non-structural proteins. These non-structural proteins are not always essential for virus growth and replication but can play an important role in viral pathogenesis through their interaction with the host innate immune system. In this review, we will summarize current knowledge and understanding of insect-borne bunyavirus non-structural protein function(s) in vertebrate, plant and arthropod.

## 1. Introduction

Some of the most intriguing and important emerging and re-emerging viruses belong to the *Bunyaviridae*, a family that comprises over 350 isolates. The family is divided into five genera: *Orthobunyavirus*, *Phlebovirus*, *Nairovirus*, *Hantavirus* and *Tospovirus*. All of these viruses are pathogens of humans and animals, except for tospoviruses, which are plant pathogens. With the exception of hantaviruses, bunyaviruses are arthropod-borne viruses (so-called arboviruses) and can be transmitted by a multitude of arthropod vectors that include insects such as mosquitoes, midges and thrips (in the case of plant-infecting tospoviruses) and also arachnid ticks. Although, arboviruses of relevance to human and animal health are also found in a number of other families such as the *Flaviviridae*, *Togaviridae* and *Reoviridae* [1,2], bunyaviruses have a particularly prominent position given the number of important pathogens in this family.

Rift Valley fever virus (RVFV; *Phlebovirus*) is one of the most prominent and well-studied bunyaviruses [3]. The virus has spread from Sub-Saharan Africa to Egypt, Saudi-Arabia and Yemen and there is considerable concern that it may spread into other areas (for example Europe) where potentially competent mosquito populations are found. The virus has a significant impact on animal health but also infects humans and can cause severe disease [4]. Outbreaks can have devastating effects on the local economy through restrictions on animal trade. From a European perspective, recent events have shown that outbreaks of bunyavirus infections can occur unexpectedly. In 2011, Schmallenberg virus (SBV; *Orthobunyavirus*) was discovered in cattle near the German town of Schmallenberg [5]. The virus causes fever, diarrhea and a reduction in milk yields in ruminants, as well as malformations in calves, newborn lambs and goat kids. Since then, SBV has been detected in over 5,000 farms across Europe [6]. The virus is most likely transmitted by midges [7]. With regard to tick-borne bunyaviruses, Crimean-Congo hemorrhagic fever virus (CCFHV; *Nairovirus*) has recently emerged in Turkey and Greece [8] while human pathogenic tick-borne phleboviruses were recently discovered in China [9,10,11] and in the U.S. [12] demonstrating why bunyaviruses are the prototype emerging and re-emerging viruses. Tomato spotted wilt virus (TSWV) is an important crop pathogen that can be found on all six continents and is known to infect more than 900 species. TSWV infection can have a substantial economical impact through yield and quality losses [13].

**Figure 1:**
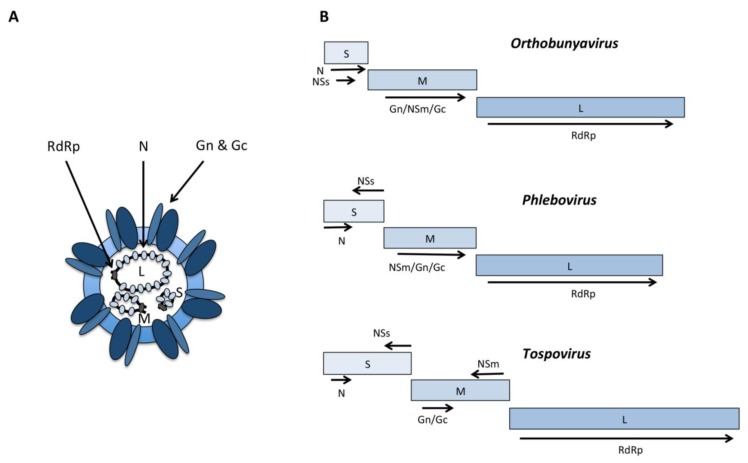
Virion structure and genetic organization of bunyaviruses. (**A**) The virion containing the three RNA segments (L, M and S) encapsidated by the N protein. (**B**) Genetic organization of the three bunyavirus genera discussed in the paper. Arrows depict the position of open reading frames (arrows below box, open reading frames in mRNAs transcribed from genome; arrows above box, open reading frames in mRNAs transcribed from antigenome).

Work on general features of bunyavirus genetics and replication has been reviewed [14], and only the key features shall be summarized here. The family is characterized by a three-segmented, negative sense RNA genome. The segments are named according to size L (large), M (medium) and S (small). A common feature of all bunyaviruses is that the L segment encodes an RNA-dependent RNA polymerase (RdRp), the M segment encodes the precursor to glycoproteins Gn and Gc, while the S segment encodes the nucleocapsid protein N. Genomes are encapsidated by the N protein, and complementary sequences at the 3’ and 5’ genome termini give the segments their characteristic circular “panhandle” structure (Figure 1(A)). Our understanding of bunyavirus biology and protein/gene functions has been much advanced through the use of modern molecular biology and the development of reverse genetic systems. In addition to the proteins mentioned above, non-structural proteins encoded by bunyaviruses (Figure 1(B)) have received much attention due to their ability to interact with the vector/host immune system and contribute to the viral pathogenesis. This review will focus on these non-structural proteins within the different genera of insect-borne bunyaviruses, specifically orthobunyaviruses, phleboviruses and tospovirus, and outline recent advances on their functions and modes of actions. Although members of the *Nairovirus* genus also are arthropod-borne (with ticks as vectors) and CCHFV has been described to produce a non-structural protein [15], there is no knowledge of its function and this genus will not be discussed further in this review.

## 2. Genus Orthobunyavirus

The *Orthobunyavirus* genus contains over 170 viruses, grouped into 48 species with Bunyamwera virus (BUNV) being the prototype virus both for the genus as well as for the whole family [16]. The genus consists of many important pathogens of both humans and animals, such as La Crosse virus (LACV) causing encephalitis in children, Akabane virus (AKBV) causing congenital malformations in ruminants and SBV.

### 2.1. The NSs Protein of Orthobunyaviruses

The availability of reverse genetic systems for BUNV, LACV, AKBV [17,18,19] and more recently SBV [20,21] have revolutionized our understanding of bunyavirus biology, and have significantly increased the knowledge of the role(s) of the non-structural proteins. The NSs proteins of orthobunyaviruses, which are 83 to 109 amino acid residues in length [22], are encoded from the +1 open reading frame (ORF) within the N ORF on the S segment (Figure 1(B)), which allows for mutations to be introduced that delete the NSs ORF while N is translated. These NSs proteins have been found to be not essential for virus growth and can be deleted, but contribute to viral pathogenesis [23,24]. BUNV lacking NSs (∆NSs) has a reduced growth rate as well a reduced ability to shut off mammalian host cell protein synthesis compared to wildtype (wt) BUNV [23]. A similar effect is observed comparing wt LACV to LACV∆NSs [17]. Furthermore, *in vivo* mouse experiments demonstrated that although the tropism of the virus in the brain is the same between wt BUNV and BUNV∆NSs, wt virus replicates and spreads faster than the mutant virus [23]. Similarly, deletion of NSs clearly attenuates the virus in infected mice and thus acts as a virulence factor [18]. In *Aedes albopictus*-derived mosquito cells (C6/36), however, BUNV NSs does not inhibit protein synthesis [24,25]. Also, overexpression of LACV NSs in mammalian 293T cells suppressed RNA interference (RNAi) in these cells [26], while LACV NSs as inhibitor of RNAi in insect cells (*Aedes albopictus* U4.4) has been ruled out [27]. Thus, the role/effect of NSs may differ in mosquito cells compared to that in mammalian cells, however these contradictory results could also be due to differences in experimental setup. RNAi is the major antiviral response in insects. Virus replication induces double-stranded RNA, which in the exogenous RNAi pathway is cleaved by the dicer-2 enzyme into small interfering RNAs, and mediates cleavage of target RNA through the RNA-induced silencing complex. BUNV NSs has been shown to be important for infection of *Ae. albopictus* U4.4 and *Ae. aegypti* Ae cells but not for infection of *Ae. albopictus* C6/36 and C7-10 cells [28]. Whether this can be linked to deficiencies in Dicer 2-based RNAi pathway [29] in the latter two cell lines is not clear. Infection of *Ae. aegypti* mosquitoes showed that BUNV∆NSs had a delayed infection progress, but when the virus managed to cross the midgut barrier it was able to spread throughout the mosquito, including the salivary glands [28]. Thus, BUNV NSs does, at least in some mosquito cell lines as well as in *Ae. aegypti* mosquitoes, appear to have a role in modulating the efficiency of viral replication.

Although, the best characterized viruses within the genus encode for the non-structural protein NSs this is not true for all orthobunyaviruses. Genetic characterization of viruses in the Anopheles A, B and Tete serogroup showed that these viruses do not encode a NSs protein and most of them behave similarly to BUNV and LACV mutants lacking NSs, in that they induce interferon-β (IFN-β) mRNA and IFN protein production. However, the one exception Tacaiuma virus (TCMV), which despite lacking NSs inhibited IFN induction through an at present unknown alternative mechanism [30]. Interestingly, Brazoran orthobunyavirus which was recently discovered in Texas, USA, appears to employ an unusual NSs expression strategy in which the NSs ORF precedes N. Whether this NSs, which with 20 kDa would be larger than other NSs proteins, has comparable functions to other orthobunyavirus NSs proteins is not yet known [31]. Thus, questions remain on the evolution of NSs proteins, and also about the mechanisms with which orthobunyaviruses use to propagate in nature, in the absence of NSs.

#### 2.1.1. Orthobunyavirus NSs Proteins and Interference with IFN α/β Induction

**Figure 2:**
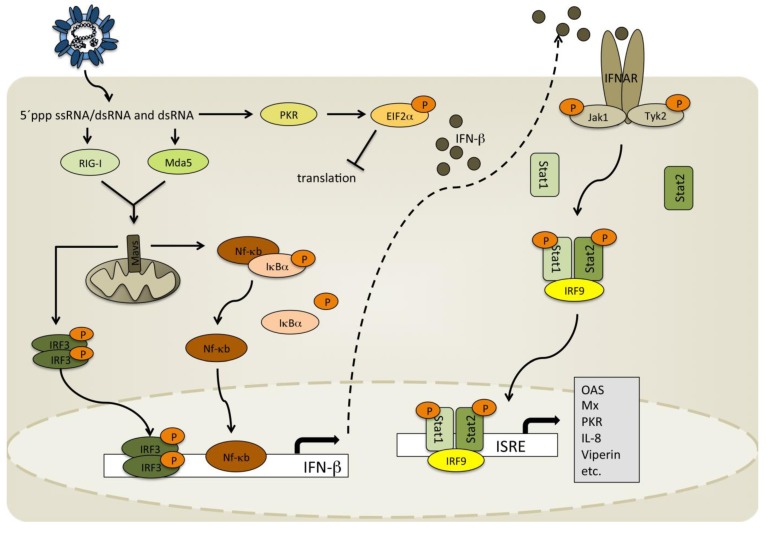
General activation of interferon-β (IFN-β) transcription and induction of IFN-stimulated genes. Biochemical and structural features in the RNA genome or dsRNA produced through viral replication is recognized by RIG-I and Mda5 in the cytoplasm leading to the activation and translocation of IRF-3 and NF-κB into the nucleus where they are involved in initiating the transcription of IFN-β. In addition, 5’-triphosphorylated dsRNA in nucleocapsids triggers the activation of RIG-I and its downstream signaling, as has been shown for Rift Valley fever virus (RVFV) and La Crosse virus (LACV) [45]. The resulting IFN-β is transported out of the cell where it binds to IFN receptors (IFNAR), thereby activating the JAK/STAT pathway leading to the transcription of a number of different IFN stimulated genes that aim to restrict viral replication and spread and initiate an antiviral state in uninfected cells.

**Figure 3:**
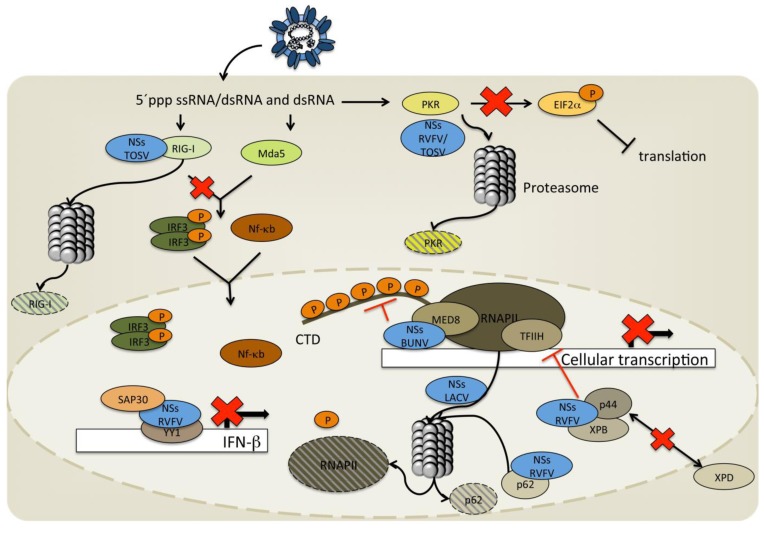
The mechanisms by which NSs of orthobunyaviruses and phleboviruses interfere with the host innate immune system. The NSs proteins of the different bunyaviruses display a variety of different mechanisms to interfere with the host antiviral defense. This is done both by inducing proteasome degradation of proteins such as PKR and RIG-I as well as interfering with the assembly and activation of the transcription machinery inducing a host cell transcription block and inhibiting IFN-β transcription.

Knowledge of the interferon pathways of vertebrates and their role in innate antiviral defenses is increasingly detailed. Virus antagonism of these host responses is recognized as a key element in virus/host interactions [32]. The induction of IFN production in virus infected cells is dependent on the activation of different factors, such as the IFN regulatory factor 3 (IRF-3) and nuclear factor κB (NF-κB) that upon activation translocate from the cytoplasm into the nucleus and there are involved in initiation of IFN induction (Figure 2) [32,33]. The crucial role of orthobunyavirus NSs proteins in counteracting host responses is now well established. Indeed, several studies have shown that the actions of BUNV, SBV and LACV NSs proteins inhibit the production of IFN in infected cells (Figure 3) and if the NSs ORF is deleted in these viruses they display an attenuated phenotype both *in vivo* and *in vitro* [21,23,27,34]. In the brain LACV and LACV∆NSs replicate mainly in neurons, however, these infected neurons are not the major contributors to IFN-β production [35,36]. During LACV infection uninfected astrocytes and microglia are the main producers of IFN-β, while in infections with LACV∆NSs astrocytes are the dominant IFN-β producer. LACV NSs strongly inhibits IFN-β production in astrocytes while it does not affect the amount of IFN-β produced in microglia [36]. Much of the pioneering work on understanding how orthobunyaviruses inhibit induction has been carried out with BUNV. As discussed in more detail in Section 2.1.2, IRF-3 is activated by BUNV and BUNV∆NSs viruses to a similar extent and is shown to enter the nucleus upon infection [37,38]. Moreover, Protein kinase R (PKR) is also activated by BUNV infection, leading to the phosphorylation and activation of the α subunit of the eukaryotic translation initiation factor 2 (eIF2) and NSs is not able to inhibit this activation. Streitenfeld *et al*. (2003) demonstrated that BUNV appears not to be sensitive to the antiviral effects of PKR in cell culture but *in vivo* PKR appears to have a minor contribution to host resistance [34]. However, it should be noted that in a study overexpressing PKR in HEK 293 cells PKR did display a clear antiviral effect, reducing viral replication about 10-fold [39]. In BUNV infection inhibition of transcription must occur downstream of transcription factor activation. Chromatin immunoprecipitation analysis revealed that BUNV NSs does not interfere with the RNA polymerase II binding to the IFN-β promoter. Instead, NSs of BUNV prevents the phosphorylation of serine 2 in the heptapeptide repeats (YSPTSPS) in the C-terminal domain (CTD) of RNA polymerase II (Figure 3) [38]. This attacks a fundamental process as phosphorylation of serine 2 of CTD is needed for mRNA elongation and 3’-end processing [40]; thus by interfering with phosphorylation of this residue, BUNV can induce a generalized transcriptional block including that of IFN-β gene transcription. However, BUNV NSs does not target CTD phosphorylation in insect cells, which may contribute to the establishment of persistent infection in these cells [38]. This inhibition of phosphorylation and consequently of transcription is believed to be mediated by the interaction between the C-terminus of BUNV NSs and the protein Med8 [41]. Med8 is a component of the Mediator complex, which regulates RNA polymerase II through interacting directly with the CTD [42]. Although the interaction of the C-terminus of NSs with Med8 is required [41], van Knippenberg *et al*. (2010) showed that the N-terminus of the NSs protein is also involved in transcription inhibition [43]. LACV triggers IFN induction through the RIG-I pathway and subsequently activation of IRF-3, and similar to BUNV, LACV virus targets RNA polymerase II in order to inhibit IFN transcription. This inhibition is not mediated through interaction with Med8 or inhibition of phosphorylation of CTD but through elimination of the hyperphosphorylated (IIo) form of RNA polymerase II via a proteasomal degradation pathway (Figure 3). NSs uses the DNA damage response (DDR) pathway to trigger the degradation of CTD-Ser-2 phosphorylated RNA polymerase II, which leads to transcriptional arrest [44]. Thus, NSs proteins are important IFN antagonists. However, incoming viruses are not resistant to the antiviral effects of IFN as treatment of cells with IFN prior to virus infection significantly reduces virus yield [34]. BUNV interferon-induced gene MTAP44 and in particular Viperin appear to be involved in restricting BUNV replication [39].

#### 2.1.2. Apoptosis

Induction of apoptosis is a common defense strategy of the host to stop dissemination of virus [33]. Upon viral infection the ubiquitously expressed protein IRF-3 is activated through phosphorylation, leading to its homodimerization and translocation to the nucleus where, in addition to activating transcription of IFN, a number of other antiviral genes that may induce apoptosis are activated [46,47]. Following virus infection, IRF-3 has also been shown to interact with the pro-apoptotic protein Bax in the cytoplasm and to translocate to the mitochondria triggering the mitochondrial intrinsic apoptotic pathway [48,49]. It is therefore not surprising that many viruses have evolved counter measures to prevent the induction of apoptosis. The NSs protein of BUNV has been suggested to have anti-apoptotic activity in mammalian cells as BUNV∆NSs virus induces cell death earlier than wt BUNV [24,37]. As IRF-3 activation and nuclear translocation is similar between cells infected with wt BUNV and those infected with BUNV∆NSs, NSs must suppress events downstream of IRF-3 [37]. The mechanism for this is at present unknown. Unlike BUNV NSs delaying apoptosis, LACV NSs has been shown to induce apoptosis [17,50]. The NSs protein of LACV, and of other members of the California serogroup (San Angelo virus and California encephalitis virus), contains a suggested 59 amino acid reaper-like region (RLR) in the C-terminus [50]. Reaper is a *Drosophila* protein with an established ability to induce apoptosis by binding to inhibitor of apoptosis proteins (IAPs) and thereby block the IAPs from binding and regulating caspases [51]. Like Reaper, San Angelo virus (of the California serogroup) NSs protein was shown to inhibit protein translation as well as to induce apoptosis upon expression of NSs. The induction of apoptosis was suggested to occur by a similar mechanism to that of Reaper as NSs was shown to interact with the same apoptotic regulator protein, Scythe [50]. Thus, differences between NSs proteins also appear to give different biological activities.

### 2.2. The NSm Protein of Orthobunyaviruses

Apart from NSs, orthobunyaviruses also encode the non-structural NSm protein on the M segment (Figure 1). In the case of BUNV, NSm is situated between the glycoproteins Gn and Gc in the M segment polyprotein precursor (1,433 amino acids), and consists of amino acids 303 to 477 that are released from the polyprotein following proteolytic cleavage. The protein is predicted to consist of three hydrophobic and two non-hydrophobic domains. Two of the NSm domains are non-essential and this property has been used to insert green fluorescent protein into the M segment and subsequently rescue a recombinant fluorescent virus [52]. Little is known about the exact function of NSm. However, the N-terminus of the NSm protein has been shown to be required for viral assembly [52]. Indeed, localization studies have shown its presence in Golgi-derived tubular structures, which house replication complexes that act as virus factories. Therefore, it was suggested that NSm may act as a scaffolding protein [53]. No further roles for these NSm proteins have yet been identified and if it has a role in mosquito infection it is at present unknown.

## 3. Genus Phlebovirus

The Phlebovirus genus comprises of nine species, with the majority of them being transmitted by sandflies (*Phlebotomus*) but also some using ticks and mosquitoes as vectors [54]. The best studied virus within this genus is RVFV [3,55], although work on the molecular biology and structure of phleboviruses has also been carried with Uukuniemi virus. Among other important pathogens in the family is Toscana virus (TOSV), which causes meningitis and encephalitis in the Mediterranean areas [56]. More recently, newly discovered phleboviruses are the tick-borne severe fever with thrombocytopenia syndrome virus (SFTSV) found in China [11] and Heartland virus discovered in Missouri, USA [12].

### 3.1. The NSs Proteins of Phleboviruses

The NSs proteins of phleboviruses are encoded in antisense orientation on the S segment (Figure 1). The best studied NSs protein is that of RVFV. It is known to form filamentous structures in the nuclei of mammalian cells after infection. Filament formation is mediated by oligomerization of the carboxy- terminal domain of the protein due to phosphorylation by casein kinase II at two serine residues located in the carboxy terminus [57]. These filaments generally appear to be mostly separate from cellular DNA; however heterochromatin satellite clusters have been associated with NSs filaments, thus likely resulting in a high incidence of nuclear anomalies and known chromosome cohesion and segregation defects [58]. RVFV NSs interacts with a number of different cellular promoter regions and thereby possibly regulates the expression of each specific targeted gene. NSs does not only interact with promoters of the innate-immunity regulating genes such as IFN-β, and those involved in inflammation, IL10Rb, but also with the promoters of a number of genes involved in coagulation, cell adhesion and neurological functions. The effect on the expression of these genes can help us to further understand the pathological effects seen upon RVFV infection [59].

Lack of functional NSs abrogates RVFV competency to replicate in type I IFN-competent cells and results in the attenuation of RVFV in animals, suggesting that NSs is a major virulence factor of RVFV [60,61]. More recently, the NSs protein of TOSV was also identified as an interferon antagonist [62,63]. Thus, even though NSs is not essential for viral replication *per se*, it is important in the context of virus/host interactions. The best investigated cellular pathways targeted by NSs, described in detail below, include those where NSs specifically target the IFN α/β pathway and those that lead to a general shut down of host transcription [14]. The role of NSs in arthropod infection is not known, but RVFV NSs does not affect the infection or transmission rate in either *Ae. aegypti* or *Culex quinquefasciatus* mosquitoes. The dissemination rate, however, is somewhat decreased in *Ae. aegypti* mosquitos when infected with RVFV lacking NSs [64]. It has also been shown that RVFV NSs forms filaments not only in mammalian cells but also in the nucleus of the mosquito cell lines Aag2 and C6/36, but not in U4.4 cells. In Aag2 cells the filaments could be observed early in infection (24 h) but completely disappeared later in the infection. However, in C6/36 the filaments were also seen at early time points but unlike the Aag2 they remained throughout the infection period [65].

#### 3.1.1. Mechanisms for Inhibition of Host Transcription by NSs

As for the orthobunyaviruses, the NSs proteins of phleboviruses are virulence factors whose best known function is IFN induction antagonism. The NSs protein of RVFV has been shown to be able to inhibit IFN-β expression without interfering with the activation of the three main transcription factors IRF-3, AP-1 and NF-κB [66]. The inhibition of IFN and host transcription by phleboviruses occurs by several mechanisms (Figure 3). In RVFV infected cells NSs inhibits IFN induction as early as 3–4 h post infection (p.i.) by a specific interaction with SAP30 [67]. SAP30 is a component of the Sin3A/NCoR/HDAC repressor complex [68] and has been shown to interact with the transcription factor YY1 [69]. YY1 suppresses IFN-β expression through direct interaction with the IFN-β promoter [70]. The interaction between SAP30, NSs and YY1 binding to the IFN-β promoter (-90 position) leads to the formation of a protein complex that despite activated IRF-3 in the nucleus inhibits the recruitment of CBP and histone acetylation and thereby avoids IFN-β transcription (Figure 3) [67]. Interestingly, TOSV NSs interferes with the production of IFN through targeted activation of IRF-3 [63]. This occurs through a RIG-I dependent signaling pathway where interaction between the NSs protein and RIG-I leads to a proteasome-mediated degradation of RIG-I [71]. TOSV NSs was also shown to degrade PKR, thus making it a versatile host response antagonist [72]. RVFV NSs also triggers degradation of PKR via a proteasome-dependent pathway [73,74] leading to the inhibition of eIF2a phosphorylation thereby facilitating viral translation [74] (Figure 3).

RVFV NSs inhibition of general host transcription also occurs early following infection. Inhibition coincides with the formation of the NSs nuclear filamentous structures as well as a decrease in the cellular concentration of TFIIH (4–8 h p.i.) [75]. TFIIH is an essential transcription factor for host RNA polymerases I and II and is composed of 10 subunit proteins (XPD, p8, p34, p44, p52, p62, XPB, MAT1, cyclin H, and cdk7) [76,77]. RVFV NSs is known to suppress transcription of host mRNA through interactions with p44 as well as with the p44/XPB complex. The filamentous structures formed by the NSs protein in the nucleus co-localize with p44 and XPB. By binding to p44, NSs outcompetes XPD (the natural partner to p44) and thus disturbs the assembly of TFIIH, resulting in the inhibition of cellular transcription (Figure 3). The interaction between p44 and NSs is probably the reason behind the accumulation of XPD in the cytoplasm around the nuclear membrane [75]. Thus, NSs does not have the ability to inhibit the TFIIH activity nor dissociate the entire TFIIH but rather prevents the assembly of its subunits through binding to p44 and thereby limits the TFIIH concentration leading to an inhibition of cellular transcription. Moreover, p62 interacts with NSs and leads to proteasomal degradation of this host protein directly in the nucleus (Figure 3) [78]. Not all phleboviruses are able to affect the general host transcription. TOSV, unlike RVFV, does not appear to have this ability and must therefore rely on alternative strategies for efficient replication and spread [72].

#### 3.1.2. DNA Damage Response and p53 Activation

A number of different viruses including LACV induce the DNA damage response (DDR) pathway to aid their replication [44]. Once DNA damage occurs, major signaling checkpoints in the DDR are activated, resulting in the initiation of cell cycle checkpoints which freeze the cell cycle either at G_1_/S, intra-S phase, or the G_2_/M phase, allowing for repair or induction of apoptosis. Although RVFV does not appear to induce DNA damage, it activates phosphorylation of a number of DDR signaling proteins. Upon infection phosphorylation of ATM and of its substrates p53, Chk.2 and H2A.X are observed while reduction in phosphorylation is seen of ATR [79]. ATM and ATR are the two major kinases controlling the DDR signaling pathway [80]. The virulent wildtype ZH548 RVFV strain and attenuated vaccine strain MP-12 [81] both induce cell cycle arrest, however, interestingly in two different phases. While ZH548 arrest in Go/G1-phase, the MP-12 induces a S-phase arrest. Knock out of ATM or Chk.2 results in a lack of arrest and a reduction of viral replication is observed. A similar effect is observed when using a virus lacking NSs [79]. Thus, the ability to induce the DDR pathway is NSs-dependent and important for viral replication. As already mentioned, p53 is activated by phosphorylation upon RVFV infection and localizes into the nucleus. The observed activation of p53 leads to the up-regulation of p53 regulated genes involved in apoptosis and cell cycle control. In fact, by reducing or knocking out p53 less cell death as well as a reduction of RVFV is observed [82].

### 3.2. The NSm Proteins of Phleboviruses

The NSm proteins of this genus are also produced by cellular cleavage of the M segment polyprotein precursor. Apart from NSm, an additional nonstructural protein is encoded by the M segment—the 78-kDa protein. This protein is translated from the first start codon of the segment and includes the entire NSm and Gn [83]. The biological function of this protein is not known and it has been shown that neither the 78-kDa protein nor the NSm of RVFV is essential for viral infection or growth in cell culture [84,85,86]. However, viruses lacking NSm display an attenuated nature compared to virus expressing NSm [87]. Interestingly, studies on apoptosis have given insights on the roles of the NSm proteins. RVFV MP-12 infection leads to a more extensive induction of cell death than that of the mutant virus with a large deletion in the pre-Gn-region thus expressing neither the NSm protein nor the 78-kDa protein. Infection with this mutant leads to up-regulation of the caspase-3/7, -8 and -9 activity (caspases are effector proteases in apoptosis). NSm -contrary to the 78-kDa protein- was able to suppress caspase-3 activity and thereby inhibit apoptotic cell death. It was also shown that NSm could suppress staurosporine (STP)-induced cell death by inhibiting the activation of both caspase-8 and -9 [86]. The exact mechanism of how the RVFV NSm protein exerts its anti-apoptotic activity in mammalian host cells is, however, not yet known.

Even less is known about the role of NSm in mosquitoes, however, it has been shown that deletion of RVFV NSm leads to a significant reduction of viral infection and transmission and can nearly completely abolish the infection in *Ae. aegypti*. This indicates an important role of NSm in viral mosquito infection [64].

## 4. Genus Tospovirus

The genus *Tospovirus* is the only plant-infecting genus in the *Bunyaviridae* family. As with most other genera within the family, tospoviruses are transmitted by arthropods to their plant host. In this case, thrips have been shown to be the vector [88]. Moreover, for plant-infecting viruses only tospo-, rhabdo-, reo- and tenuiviruses replicate in their insect vector [88,89,90]. Tospoviruses have a broad host range with more than 1,000 plant species as hosts, infecting plants all over the world and thereby have a large economic impact [91]. Most research has been performed on tomato spotted wilt virus (TSWV), which is the type species of tospoviruses.

### 4.1. The NSs Proteins of Tospoviruses

The NSs protein is encoded in an ambisense strategy on the S segment of tospoviruses. It has been identified as a virulence factor in both the plant host and insect vector. In plants, accumulation of the TSWV NSs protein was observed in infected leaves, forming fibrous-like structures [92]. Accumulation of high levels of NSs in salivary glands of thrips has been reported, suggesting that NSs is co-injected into plants during feeding [93].

**Figure 4:**
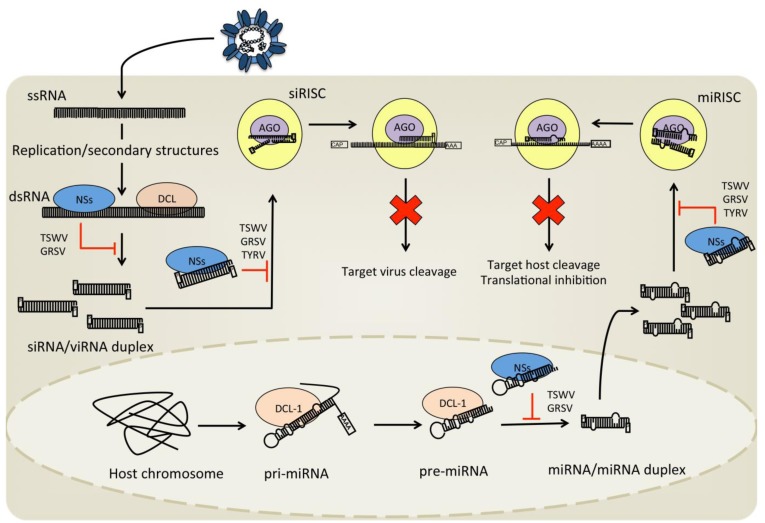
Tospovirus NSs interaction with the RNAi pathway in plants. Upon infection of tospovirus in plants siRNA/viRNA pathways are activated as a response to the viral dsRNA. This pathway activation leads to specific cleavage of viral RNA. The miRNA pathway is activated by expression of endogenous primary miRNA transcripts and can lead to translational inhibition and cleavage of host mRNA. This figure illustrates where the NSs of different tospoviruses act in order to interfere with these small RNA pathways of the host.

NSs proteins of some tospoviruses have been shown to interfere with the antiviral RNAi or RNA silencing response in plants (TSWV, tomato yellow ring virus [TYRV]) [94,95,96] and different arthropod cells (TSWV in drosophila, tick and mosquito) [27,95,97]. Similar results have been reported for the NS3 proteins of the tenuiviruses, rice hoja blanca virus and rice stripe virus, which share high phylogenetic similarities with tospoviruses and encode the NS3 ORF at an analogous position in the viral genome [94,98,99,100,101]. The RNAi response is a sequence specific breakdown mechanism, which can be divided into different pathways dependent on the small RNA molecules involved. The small interfering (si)RNA pathway is known to act antivirally in plants and arthropods [102,103]. It is induced by exogenous long dsRNA molecules that are recognized and cleaved into siRNA molecules. The siRNAs (or viRNAs) are taken up by a multi-protein complex that uses one strand of the siRNA as a guide to find complementary single stranded RNA, which is cleaved and degraded after recognition (Figure 4). In contrast, the micro (mi)RNA pathway is induced by exogenous single stranded RNAs that fold back to form a stem loop structure, and upon cleavage result in miRNA duplexes. One strand of these duplexes is used as a guide to find (partially) complementary sequences, resulting in target cleavage or translational inhibition (Figure 4). The miRNA pathway is present in a variety of organisms and used to regulate gene expression, mainly during development. The antiviral potential of the siRNA pathway is well established in plants and arthropods and, with regards to arbovirus vectors, in particular mosquitoes [104].

Biochemical analyses have shown that tospoviral NSs proteins are able to bind dsRNA molecules (of the siRNA and miRNA pathway), which are key molecules in the RNAi pathways (Figure 4). However, not all tospovirus NSs proteins investigated so far have the same affinity for different types of dsRNA molecules. The NSs proteins of the American clade tospoviruses (e.g., TSWV, ground ring spot virus [GRSV] and impatiens necrotic spot virus [INSV]) are able to bind long (dsRNA and precursor-miRNA) and short dsRNA (siRNA and miRNA duplexes) molecules with a similar affinity; in contrast to the Eurasian clade NSs (TYRV) that can only bind short dsRNA molecules [99]. A similar discrepancy between different viruses of the same genus has been reported for viruses belonging to the tombusvirus genus.

Recently, TSWV NSs has been reported as an avirulence (Avr) determinant, triggering *TSW* gene based resistance in peppers (*Capsicum annuum*). In addition, NSs of a resistance-breaker strain had lost the RNAi suppressor activity in transient reporter assays in plants, present for the resistance-inducing TSWV NSs; however, both TSWV NSs proteins were able to interfere with RNAi during a natural viral infection in plants [105]. This suggests an additional role for TSWV NSs besides the well described RNAi suppressor activity. Besides, TSWV NSs in concert with N and the hairpin structure found between the N and NSs ORF on the S segment has recently been suggested to have a role in translation [106]. More research is needed to investigate if these findings can be broadened to other tospoviruses and to investigate if the different affinities for small RNA molecules observed for the American and Eurasian clades can be linked to, for example, virulence or translational activity.

### 4.2. The NSm Proteins of Tospoviruses

Tospoviruses can be separated into two major clusters: New World and Old World, depending on phylogenetic analysis of NSm and N proteins. TSWV belongs to the New World cluster and the NSm proteins in this cluster have a hypervariable N-terminus (50 amino acids), however the central part of the NSm contains conserved regions shared between members within this group [107].

The NSm protein of TSWV has been suggested to display several activities during viral infection in the plant hosts including cell-to-cell movement, long distance movement and symptom induction. NSm has been shown to be expressed early in systemic infections and forms aggregates with the nucleocapsid in the cytoplasm of infected cells [108]. Movement proteins are essential for plant infecting viruses as they ensure the spread of the virus from the infected cell to a new uninfected cell. As plant cells have a cell wall, unlike mammalian cells, which only have a cell membrane, plant viruses need different ways to spread between cells [109]. NSm has been shown to localize to plasmodesmata [110], which results in plasma modification [110,111] and can form tubule structures if expressed in either insect cells or protoplasts [112,113]. Biochemical analysis of NSm showed its ability to bind single-stranded RNA sequence unspecifically and to interact with several host trafficking proteins [114,115]. All these properties supported the suggestion of NSm being the movement protein of TSWV. The ability of TSWV NSm to complement a movement deficient tomato mosaic virus (TMV) [112], gave further evidence for NSm being a movement protein. Besides, TMV expressing TSWV NSm showed TSWV specific symptoms in infected cells, suggesting NSm to be involved in disease symptoms [110,112]. Mutational analysis has linked the C-terminus of TSWV NSm with tubule formation and movement [112]. Further fine mapping of the central regions has identified several areas that could be linked with cell-to-cell movement and tubule formation; suggesting a correlation between these features. In contrast, long distance movement could be mapped to different regions, supporting an independent action between cell-to-cell and long distance movement. Regions linked to necrosis in infected plants are again different [107]. These results suggest that unique regions of NSm are important for different features. Recently, TSWV NSm has also been proposed to be the resistance gene for SW5 resistance against TSWV, GRSV and tomato chlorotic spot virus infection as mutations in NSm TSWV were able to overcome the resistance [116].

To date, little is known regarding the NSm proteins of other tospoviruses The NSm of INSV, also of the New World cluster, has been shown to interact with itself as well as with N and ribonucleoprotein complexes, similar to TSWV NSm; supporting its activity as movement protein [117]. No function has been linked to tospovirus NSm in the insect vector. More work is required to determine these functions in detail. Given the current knowledge of NSm proteins and their roles in inducing disease, this is likely to generate important insights into the biology of these plant-infecting viruses.

## 5. Conclusions

Members of the *Bunyaviridae* family infect a number of different hosts, and each specific virus has to be able to infect and replicate in arthropod and vertebrate or plant hosts. Non-structural proteins and in particular the NSs protein have, as reviewed above, been shown to be important virulence factors acting as host response antagonists. The exact roles of NSm proteins in assembly and possibly other functions still have to be described in more detail. Moreover, mechanisms are likely to differ between and even within genera. Even less is known when it comes to the virus-host interaction in vectors and plants, although both use RNAi as important antiviral defense mechanisms. Analyzing the protein interaction networks involving these non-structural proteins would strongly advance our understanding of the mechanisms by which these proteins exert their functions. In combination with reverse genetics approaches that allow manipulation of viral sequences, we should see an increasingly complete picture of the function of non-structural proteins and of their relevance to bunyavirus infections. New sequencing technologies allowing large-scale approaches will provide a more comprehensive overview of the different host cellular pathways that are activated upon bunyavirus infections as well as how and which of these the viruses target. Thus, research on bunyavirus molecular biology will increase our understanding of non-structural proteins and will help to guide the efforts to produce better preventive measures and control measures such as vaccines and potentially treatments. It will also allow better preparedness for novel and/or emerging bunyaviruses as methods and protocols are in place to study and manipulate those pathogens. In the future, more *in vivo* work in vertebrates as well as arthropod vectors will be required to study bunyavirus pathogenesis and transmission. With the tools described in this review, such as viruses carrying deletions *etc*., those studies will take our knowledge of bunyavirus biology to the next level. The diverse and original coding strategies that are employed by bunyaviruses to express these non-structural proteins is a clear indication of their importance, although it also opens the question on how bunyaviruses that do not appear to encode or express non-structural proteins replicate efficiently. As we characterize an increasing number of representatives from this virus family, we can expect more challenges and questions to arise.
